# Development of Rift valley fever encephalitis in rats is mediated by early infection of olfactory epithelium and neuroinvasion across the cribriform plate

**DOI:** 10.1099/jgv.0.001522

**Published:** 2020-11-24

**Authors:** Devin A. Boyles, Madeline M. Schwarz, Joseph R. Albe, Cynthia M. McMillen, Katherine J. O'Malley, Douglas S. Reed, Amy L. Hartman

**Affiliations:** ^1^​ Center for Vaccine Research, University of Pittsburgh, Pittsburgh, PA, USA; ^2^​ Department of Infectious Diseases and Microbiology, University of Pittsburgh, Pittsburgh, PA, USA; ^3^​ Department of Immunology, University of Pittsburgh, Pittsburgh, PA, USA

**Keywords:** aerosol, cribriform plate, encephalitis, olfactory bulb, pathogenesis, Rift Valley fever virus

## Abstract

The zoonotic emerging Rift Valley fever virus (RVFV) causes sporadic disease in livestock and humans throughout Africa and the Saudi Arabian peninsula. Infection of people with RVFV can occur through mosquito bite or mucosal exposure during butchering or milking of infected livestock. Disease typically presents as a self-limiting fever; however, in rare cases, hepatitis, encephalitis and ocular disease may occur. Recent studies have illuminated the neuropathogenic mechanisms of RVFV in a rat aerosol infection model. Neurological disease in rats is characterized by breakdown of the blood–brain barrier late in infection, infiltration of leukocytes to the central nervous system (CNS) and massive viral replication in the brain. However, the route of RVFV entry into the CNS after inhalational exposure remains unknown. Here, we visualized the entire nasal olfactory route from snout to brain after RVFV infection using RNA *in situ* hybridization and immunofluorescence microscopy. We found widespread RVFV-infected cells within the olfactory epithelium, across the cribriform plate, and in the glomerular region of the olfactory bulb within 2 days of infection. These results indicate that the olfactory tract is a major route of infection of the brain after inhalational exposure. A better understanding of potential neuroinvasion pathways can support the design of more effective therapeutic regiments for the treatment of neurological disease caused by RVFV.

## Introduction

Rift Valley fever virus (RVFV) is an emerging mosquito-borne zoonotic pathogen that is endemic to Africa and the Middle East, where it infects domestic ruminants and causes fatal hepatic necrosis and ‘abortion storms’ amongst livestock herds [1]. Humans can be infected via mosquito bite, or from mucosal exposure and/or inhalation of infectious particles from affected livestock [1]. Infected people typically experience a self-limiting febrile illness that, in 1–2 % of cases, can advance to haemorrhagic fever, hepatitis, neurological disease, or ocular disease [2–4]. In patients that develop encephalitis, there is a 50 % chance of death, while survivors can experience long-term neurological complications [5]. Individuals with ocular lesions may experience permanent blindness [3, 5, 6]. Tissue samples from human autopsies are limited and show brain lesions, neuronal death and inflammation within the central nervous system (CNS) [7]. Animal workers such as farmers, veterinarians and butchers are at high risk for mucosal or respiratory exposure and more severe clinical outcomes from RVFV [8–11].

There is a significant risk of death and morbidity in the event that RVF progresses to neurological disease, and not much is known about the pathogenic mechanisms of RVF encephalitis compared to the more extensively studied hepatic disease. In several animal models, respiratory infection by intranasal or aerosol exposure is more likely to lead to encephalitis than peripheral infection routes [12–18]. Our previous studies have characterized the Lewis rat model of RVF encephalitis, in which rats exposed to aerosolized RVFV succumb to meningoencephalitis within 7–8 days post-infection (p.i.) [19, 20]. By 1 day p.i., viral RNA (vRNA) can be detected at low levels in the brain along with a transient inflammatory leukocyte infiltration [19, 20]. The later stages of disease (5–7 days p.i.) are characterized by blood–brain barrier (BBB) breakdown, an influx of neutrophils, high levels of infectious virus replicating in the brain and extensive neuronal necrosis, which is similar to the histological observations made in human patient samples [5, 7, 21]. Rats become symptomatic by 5–7 days p.i. with fever, weight loss, ruffled fur, hunched posture, porphyrin staining and erratic behavioural manifestations, such as cage circling, horizontal rolling, head tilting and tremors [19].

**Fig. 1.**
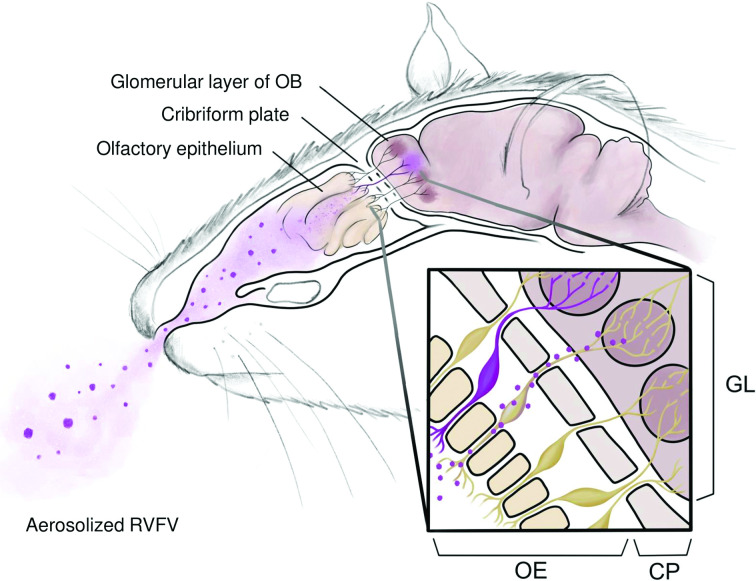
Structure of the rat nasal passages and proposed pathway of aerosolized Rift Valley fever virus neuroinvasion. Inhaled particles contact the olfactory epithelium (OE), which contains dendritic processes from olfactory receptor neurons (ORNs). Axonal projections from ORNs extend through channels in the cribriform plate (CP) and into the glomerular layer (GL) of the olfactory bulb (OB). Zoom box: RVFV-infected ORNs (purple) or virus associated with ORNs (purple dots) crossing the cribriform plate to gain access to the neuron bundles in the glomerular layer of the olfactory bulb.

Despite increased knowledge about the pathogenic mechanisms leading to RVF encephalitis in animal models, the route of initial CNS invasion has not been elucidated in the rat model. The presence of early leukocytes and low levels of vRNA in the brain at 1–2 days p.i. suggests that the virus enters the brain before widescale vascular breakdown occurs much later in infection (5–6 days p.i.) [20]. The olfactory system presents a potential avenue of CNS invasion for pathogens entering the upper respiratory tract. Olfactory receptor neurons (ORNs) are ciliated, bipolar cells residing within the olfactory epithelium (OE). Dendrites of ORNs project into the surface of the OE, whereas the axons of ORNs span into the lamina propria, extend through channels in the cribriform plate and attach to the glomerular layer within the olfactory bulb (OB) of the brain (Fig. 1). The earliest appearance of RVFV vRNA in this region at 1–2 days p.i. suggests the ability of the virus to either directly infect ORNs (as seen with herpes simplex type-1 virus [22, 23] and Nipah virus [24]) or to travel through neuron unsheathing cell channels (as, potentially, seen in La Crosse virus infection [25]). Other neuroinvasion mechanisms, such as haematogenous entry via the BBB (as seen with Ebstein–Barr virus [26]), ‘trojan horse’ entry into the BBB via infection of monocytes (as seen in simian immunodeficiency virus infection [27]), or travel through neuromuscular junctions (as seen with poliovirus [28, 29]), are less likely. The direct connection between the olfactory neurons and the OB is short, and channels formed between individual olfactory neurons can fit particles up to 100 nm. Bunyaviruses, which are 90 to 100 nm in diameter [30], could potentially pass through these channels.

To study the route of neuroinvasion after inhalation of RVFV, we sought to visualize RVFV infection of the CNS through the olfactory route. Immunostaining and RNA *in situ* hybridization (ISH) techniques allowed tracking of RVFV from the nasal epithelium, along the ORNs, across the cribriform plate, and into the OB. This study provides foundational information on the olfactory route for neuroinvasion by RVFV after aerosol infection.

## Methods

### Biosafety

All work with infectious RVFV (strain ZH501) was performed at biosafety level 3 (BSL-3) in the Regional Biocontainment Laboratory (RBL) at the University of Pittsburgh Center for Vaccine Research. Personnel wore powered air purifying respirators (PAPRs), waterproof gowns, two pairs of gloves and facility-designated scrubs and shoes. Work was conducted in a class III biosafety cabinet (BSC) using Vesphene IIse (Steris Corporation) at a 1 : 128 working dilution as a disinfectant. All tissues or samples removed from BSL-3 were inactivated using the methods described below; all inactivation methods were verified and approved by University of Pittsburgh biosafety oversight committees. The authors are approved for work with RVFV by the Federal Select Agent Program (FSAP).

### Virus propagation

The ZH501 strain of RVFV used in these experiments was generated by reverse genetics and provided by Barry Miller (CDC, Fort Collins, CO, USA) and Stuart Nichol (CDC, Atlanta, GA, USA). Virus was propagated from VERO E6 (CRL-1586, American Type Culture Collection) cells in standard culture conditions using Dulbecco’s modified Eagle’s medium (DMEM) containing 2 % (D2) or 10 % (D10) foetal bovine serum (FBS), 1 % penicillin/streptomycin (pen/strep) and 1 % l-glutamine.

### Animal infections

Female Lewis rats (8–10 weeks of age; Envigo) were infected with RVFV ZH501 by aerosol inhalation as described previously [31], with a presented dose of 3×10^4^ plaque-forming units (p.f.u.) per rat. Three rats were euthanized at 2, 5 and 7 days p.i. to collect olfactory tissues. Uninfected control rats were similarly euthanized for control tissues. Aerosol infections were performed in a class III aerobiology cabinet as described elsewhere [31].

### Whole-head tissue preparation

Rats were perfused with 500 ml of 4 % methanol-free paraformaldehyde (PFA) following euthanasia to ensure complete inactivation of tissues in the entire animal. Heads were removed and submerged in fresh 4 % PFA and stored at 4 °C for fixation for at least 1 week before removal from BSL-3 conditions according to approved inactivation protocols. Decalcification of samples was accomplished by submersion in 10 % tethylenediaminetetraacetic acid (EDTA) (pH 7.4) for 3 weeks; each week the sample was moved to a container of fresh EDTA. Decalcification with EDTA is gentle on tissues, preserves DNA and is ideal for use with immunofluorescence [32]. After decalcification, the heads were washed with 1× phosphate-buffered saline (PBS) and placed in 70 % ethanol for transport to the University of Pittsburgh McGowan Institute Histology Core for paraffin embedding and sagittal sectioning

### Fluorescence and colorimetric *in situ* hybridization and histology

Whole-snout slides sectioned at a thickness of 8 µm were deparaffinized using an alcohol dehydration series (100, 95 and 70 %; each step was repeated twice using fresh reagents and a 10 min incubation time). Slides underwent an antigen retrieval process by boiling in the RNAscope Antigen Retrieval Reagent for 15 min to unmask antigen and probe-binding epitopes. Slides for fluorescent imaging were permeabilized for 30 min using 0.1 % TritonX-100 detergent+1× PBS at room temperature (RT) (the RNAscope protease reagent step was omitted to prevent degradation of neuronal structure); colorimetric slides followed the RNAscope 2.5 HD Red detection kit procedure according to the manufacturer’s instructions with RVFV ZH501 probe against the NP gene (Advanced Cell Diagnostics cat no. 496931). Slides were stained in a slide box lined with wet paper towels and placed in an incubator at 37 °C. Fluorescent samples were stained with Neurotrace-640 (Thermo Fisher Scientific; 1 : 100 for 1 h at RT) to visualize neuronal bodies, counterstained with Hoescht, and mounted using a glycerol and polyvinyl alcohol (PVA) mixture. Colorimetric slides were counterstained with haematoxylin and mounted using Permount (Fisher Chemical). Deparaffinized slides for pathology observation followed a standard haematoxylin and eosin (H and E) staining procedure. Each staining procedure was completed on five sets of snouts, which included uninfected samples and 2, 5 and 7 days p.i. samples.

### Microscopy

Fluorescent ISH slides were imaged using the Nikon A1 confocal microscope provided by the University of Pittsburgh Center for Biologic Imaging, denoised using Nikon Elements software, and contrasted using Adobe Photoshop Elements. Colorimetric ISH slides were imaged using the Zeiss Tissue Gnostics pathology microscope provided by the Center for Biologic Imaging and white balanced using Adobe Photoshop. Pathology slides were imaged using an Olympus CX41 microscope with the Levenhuk M300 base attachment.

## Results

### Inhalational exposure to RVFV causes extensive infection of the olfactory epithelium

Our previous study found that RVFV vRNA was detectable in the OB as early as 1–2 days p.i., depending upon exposure dose [19, 20]. This finding suggests that the olfactory region plays a part in CNS infection, as the virus encounters the respiratory and olfactory epithelium during inhalation. To study the route of neuroinvasion of RVFV after inhalation exposure, adult female Lewis rats (8–10 weeks old) were exposed to small-particle aerosols of RVFV as described previously [19]. The inhaled dose was 3400 p.f.u., which, based on our previous work, is approximately 34× the LD_50_. At 2, 5 and 7 days p.i., three rats were euthanized on each day, perfused with 4 % PFA, and whole heads were decalcified and processed for staining.

**Fig. 2.**
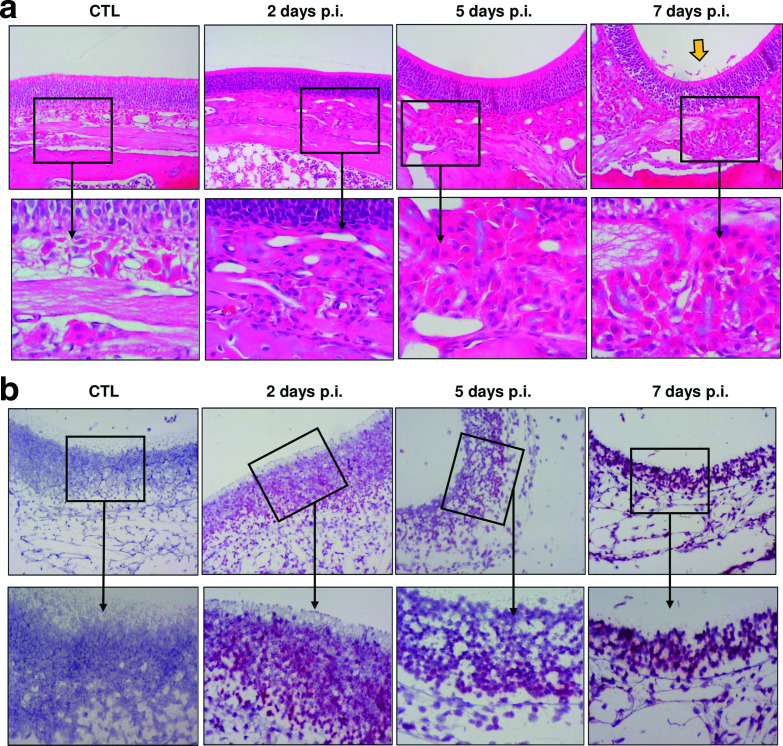
Extensive infection of the olfactory epithelium by RVFV. (a) H&E tissues at each indicated time point imaged at 10× magnification (top row) with corresponding 20× zoom boxes (bottom row). Yellow arrow indicates cilial sloughing at 7 days p.i. (b) 10× images of RNA ISH (top row) with 20× zoom boxes (bottom row). CTL represents tissue from uninfected control animals. Images shown are representative of *n*=3 at each time point.

Whole rat heads with olfactory region intact were stained with H&E to detect morphological changes within the olfactory tract. The OE remained largely intact and undamaged until 7 days p.i., when cilial sloughing was observed (Fig. 2a, yellow arrow). Extensive detection of vRNA by ISH was observed in OE as early as 2 days p.i., including sustentacular cells and ORNs residing just beneath the epithelial layer (Fig. 2b). This finding suggests that RVFV does not directly damage the OE upon viral infection, yet this region is densely populated with virus-infected cells following inhalational exposure.

### Infected cells are found in the cribriform plate soon after exposure

**Fig. 3.**
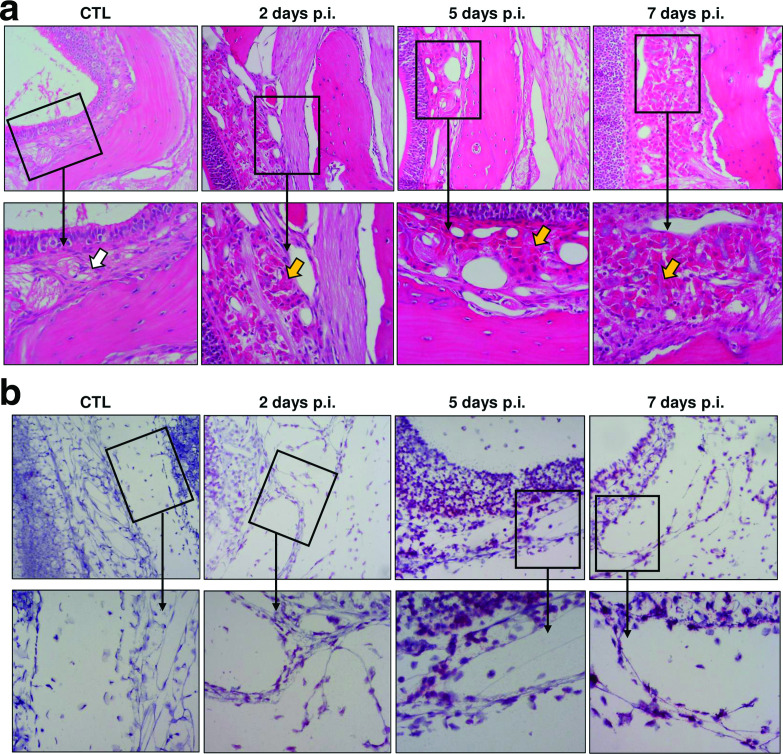
RVFV infection of cells across the cribriform plate. (a) H&E tissues at each indicated time point imaged at 10× magnification (top row) with corresponding 20× zoom boxes (bottom row). White arrow indicates lamina propria in uninfected control; yellow arrows indicate increased cellularity of this region after RVFV infection. (b) 10× images of RNA ISH (top row) with 20× zoom boxes (bottom row). CTL represents tissue from uninfected control animal. Images shown are representative of *n*=3 at each time point.

The cribriform plate is a bony structure that serves as a barrier between the nasal turbinate and brain, with small channels allowing for the passage of ORN axons from the OE into the OB (Fig. 1). These channels are essential for relaying olfactory signals from the OE to the CNS via axonal processes. Cribriform plate channels can also be exploited by pathogens and/or particulates as an entry point for access to the OB. H&E staining of the cribriform plate revealed increased cellularity in the lamina propria that lies between it and the OE (Fig. 3a, arrows). Cells within the channels of the cribriform plate stained positive for vRNA at 2 days p.i., which is evidence that this region is highly infected and likely plays an important role in viral infection of the CNS (Fig. 3b).

### Structural integrity of the glomerular layer is lost as infection progresses

**Fig. 4.**
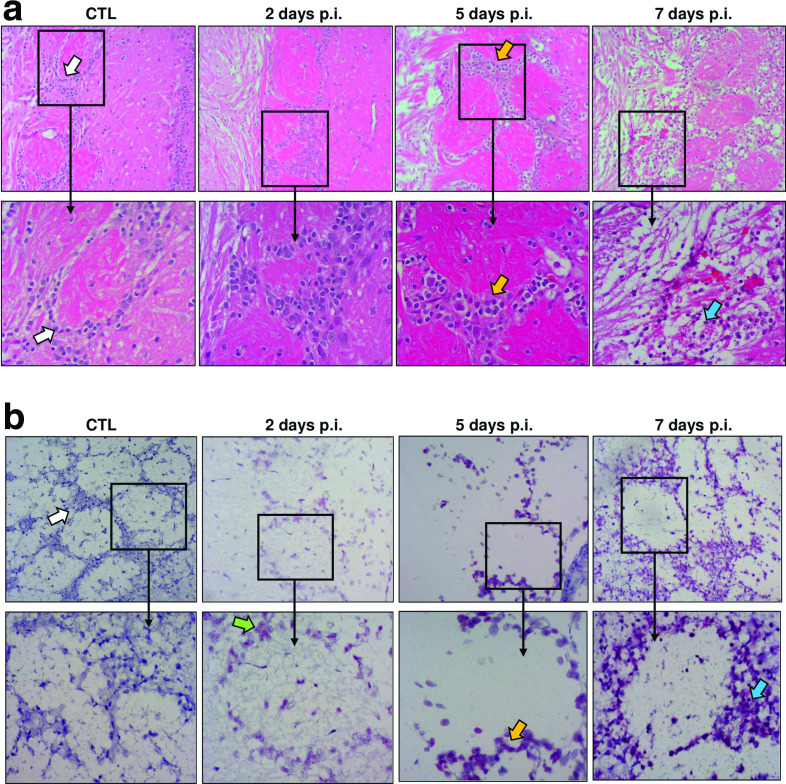
Loss of integrity in the glomerular layer of the olfactory blub as RVFV infection progresses. (a) H&E tissues at each indicated time point imaged at 10× magnification (top row) with corresponding 20× zoom boxes (bottom row). Arrows highlight periglomerular (PG) neurons forming the visible circular structure of the glomeruli. The 20× zoom box for 7 days p.i. highlights haemorrhaging. (b) Images of RNA ISH at 10× magnification (top row) with 20× zoom boxes (bottom row). In uninfected animals (CTL), the glomerular layer (GL) is intact, and healthy PG neurons are visible (white arrows). Infected PG neurons are visible at 2 days p.i. (green arrows). By 5 days p.i., the GL remains intact but PG neurons have an unhealthy appearance, concomitant with viral ISH staining (yellow arrows). By 7 days p.i., GL structure is disrupted and PG neurons are highly infected and pyknotic (blue arrows). Images shown are representative of *n*=3 at each time point.

The glomerular layer (GL) of the OB is the destination for ORN axons after crossing the cribriform plate (Fig. 1). Healthy normal GL structure is visible by distinct circular structures containing the connections between the termini of the ORN axons and the dendrites of heterogenous neuronal and glial populations surrounding the glomerulus (Fig. 4a; CTL panels). Periglomerular (PG) neurons form the circular structure that is distinctly visible (Fig. 4a, white arrows). The ORN axons meet groups of neurons in each glomerulus to relay the acquired signals to the rest of the brain. RVFV infection results in distinct morphological and structural damage in the GL, such as haemorrhage, neuronal loss and major disruption of the GL architecture (Fig. 4; 7 days p.i.) [19]. PG neurons positive for vRNA are visible at 2 and 5 days p.i., with some morphological changes in PG neuronal soma at 5 days p.i. (Fig. 4, green and yellow arrows). By 7 days p.i., the distinct boundaries of the GL become disrupted, eliminating the defined borders separating it from other layers of the OB, and the PG neurons are unhealthy with pyknotic appearance (Fig. 4, blue arrows). This structural loss of neurons and change in GL morphology coincides with the increase in vRNA visualized with ISH (Fig. 4b), as well as clinical signs of neurological distress [19].

### Inhalational exposure results in rapid infection of the olfactory bulb through the cribriform plate

**Fig. 5.**
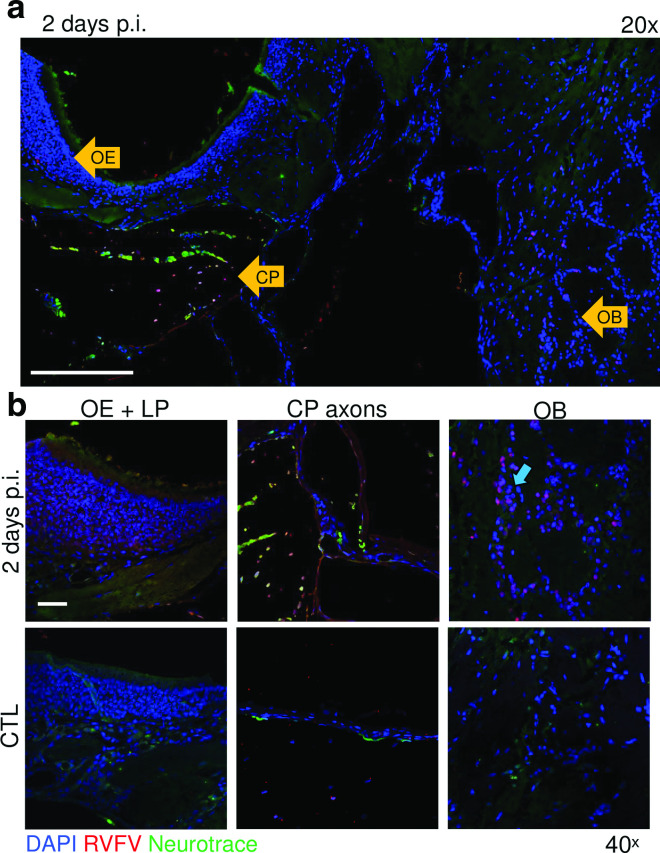
RVFV-infected cells throughout the olfactory route and olfactory bulb at 2 days p.i. (a) Image (20×) of rat snout/brain at 2 days p.i.; vRNA (red), neurons (green), nuclei (blue). Scale, 250 um. (b) Micrographs (40×) of relevant structures highlighted by yellow arrows. Blue arrow highlights infected periglomerular (PG) neurons forming the glomeruli. CP, cribriform plate; LP, lamina propria; OB, olfactory bulb. Scale, 50 um.

If RVFV can either infect ORNs or travel along them without directly infecting them, then this could account for the rapid viral invasion in the CNS after inhalational exposure. To obtain an overview of RVFV infection of the OE–OB axis early after infection, whole rat snouts were stained for RVFV vRNA by ISH along with the neuronal body dye neurotrace (Fig. 5). At 2 days p.i., RVFV-infected cells were visualized within the OE and the lamina propria, and along axons crossing over the cribriform and into the OB (Fig. 5a). Within the cribriform plate at 2 days p.i., RVFV vRNA, neurotrace and DAPI colocalized (visible by pink/orange cells), which supports the hypothesized association between RVFV and the axons of the ORNs crossing through (Fig. 5b, middle panel). A high frequency of PG neurons in the glomerulus of the OB also stained positive for vRNA at 2 days p.i. (Fig. 5b, blue arrow).

## Discussion

While mosquitos facilitate the infection and spread of RVFV between livestock, wild ruminants and humans, exposure to aerosolized RVFV has been documented. For example, abattoir workers inhale aerosolized virions during butchering of infected livestock [33–35]. Cases of respiratory exposure to RVFV have occurred among laboratory workers and in some natural outbreaks [36]; however, there is a lack of epidemiological data to directly link exposure route with severe disease outcomes. The infectiousness of RVFV by aerosol requires it to be studied within BSL-3 containment requirements, under negative pressure, and while using powered air purifying respirators (PAPRs). Aerosol exposure may be associated with a higher risk of severe human disease outcomes such as meningoencephalitis or ocular disease [5, 6, 8]. Aerosol infection studies in rats [19, 20, 31, 37], mice [16–18] and non-human primates (NHPs) [12–14, 38] result in encephalitis and higher mortality rates after aerosol infection than other exposure routes. Additional research into the effects of aerosolized RVFV is crucial because of its environmental stability and ability to be infectious via inhalation [39–41].

A large case study of 683 individuals with laboratory-confirmed RVF during the Saudi outbreak in 2000 identified the most common symptoms as fever (93 %), CNS manifestations (17 %), haemorrhagic manifestations (7 %) and vision loss (2 %) [5]. Of the 81 individuals with CNS involvement, symptoms included confusion, lethargy, disorientation, vertigo, coma, tremors and amnesia. Individuals with CNS manifestations were 14.2 times more likely to die than those without a neurological component [5]. While human autopsy specimens are difficult to obtain, a RVFV-seropositive individual who succumbed to meningoencephalitis showed pathological signs of perivascular cuffing and round-cell infiltration [21]. Similar lesions are also seen in the brains of NHPs exposed to aerosolized RVF [12].

Animal models of encephalitic RVF are crucial research tools for illuminating the mechanism of infection that separates cases of self-limiting febrile disease from severe CNS outcomes. When exposed to infectious aerosols of RVFV, Lewis rats develop lethal encephalitis within 7–8 days [31], and we have previously used this model to dissect the neuropathogenic mechanisms of RVFV infection [19, 20, 31, 42]. Our previous work illustrated the temporal spread of leukocyte infiltration and virus replication within the brain of infected rats; however, a major unknown has been the timing and route of entry of RVFV into the CNS after aerosol infection. This study was designed to address this gap in knowledge by visualizing the entire nasal olfactory route from snout to brain after RVFV infection. We optimized several techniques to accomplish this, including proper fixation, decalcification, staining and visualization of viral infection and structural markers.

The structure of the olfactory region in rats is illustrated in Fig. 1. It should be noted that the nasal cavities of human and rodents differ. Human nasal turbinates are relatively simple and prioritize breathing, while rodent nasal turbinates fold and branch in a complex manner that prioritizes olfaction and protection of the lower respiratory tract [43]. In order to support this acute olfaction, rodents have a larger surface area of OE than primates (4 and 1.25 %, respectively [44, 45]). In both rodents and primates, ORN bodies reside within the OE, with dendrites that extend down into the OE surface. In humans, the axons of over 10 million ORNs in the OE extend up into the lamina propria, across the cribriform plate, and into the OB. The GL of the OB is the point of attachment of ORN axons into the brain. Glial ensheathing cells support the axons of ORNs as they cross the cribriform plate. These ensheathing cells form fluid-filled channels that remain open and accessible to pathogens, even after ORNs degrade from age. Viral pathogens (influenza viruses [46], herpesviruses [47] and poliovirus [28, 48]) are suspected to use the olfactory route for CNS entry in rodents, NHPs and humans. La Crosse virus, another bunyavirus, was also found to use the olfactory route to enter the brain in mouse models [25].

The severity of RVFV infection in the rat model was evident based on the abundance of infected cells within the OE, the cribriform plate and the glomeruli of the OB by 2 days p.i. Of note is the finding that the OE shows little to no structural damage, unlike what is seen with other neuro-invasive pathogens, such as *Neisseria meningitidis* [49], *Listeria monocytogenes* [50], vesicular stomatitis virus [51], influenza [46] and coronaviruses such as severe acute respiratory syndrome coronavirus 2 (SARS-CoV-2) [52]. We noted only minor sloughing of the cilia late in infection when the animals display clinical signs and have massive leucocyte inflammation and tissue damage in the CNS [19].

ORNs have the ability to uptake exogenous materials and transynaptically transport them to the rest of the CNS; therefore utilization of these neurons could allow RVFV access to the rest of the brain [53]. Additionally, because the OB forms direct connections with the frontal cortex and bypasses the thymus, olfactory pathways do not undergo the antigen processing that the other sensory modalities experience [53]. The rapid appearance of infection in the OB supports the hypothesis that initial viral entry of the CNS is likely due to viral infection traversing the cribriform plate into the OB. Indeed, using immunofluorescent microscopy, we found ORN axons within the cribriform plate area colocalized with vRNA and Neurotrace at 2 days p.i. The staining used in this study cannot conclusively determine whether virus directly infects ORNs or travels through neuron unsheathing cell channels. Bunyaviruses are small (>100 nm) and could fit into these channels, which leaves the possibility for RVFV virions to enter the brain without directly infecting ORNs [54]. However, the extensive vRNA staining of cells within the OE suggests that it is highly likely that the ORNs are directly infected.

Although there are notable structural differences between the nasal mucosa of rodents and humans, the observations made in this study can help illuminate the mechanism of infection that separates disease outcome severity between subcutaneous and inhalational or intranasal exposure to RVFV. In cases of neurological involvement, RVFV leads to long-term morbidity and even death in affected individuals, and there are no current therapeutics to mitigate this disease manifestation. A more thorough understanding of potential neuroinvasion pathways can support the design of more effective therapeutic regimens.
